# Risk and Surveillance of Cancers in Primary Biliary Tract Disease

**DOI:** 10.1155/2016/3432640

**Published:** 2016-06-19

**Authors:** Valery Hrad, Yoftahe Abebe, Syed Haris Ali, Jared Velgersdyk, Mohammed Al Hallak, Mohamad Imam

**Affiliations:** ^1^Department of Internal Medicine, University of North Dakota, Fargo, ND 58102, USA; ^2^Department of General Internal Medicine, MD Anderson Cancer Center, Houston, TX 77030, USA

## Abstract

Primary biliary diseases have been associated in several studies with various malignancies. Understanding the risk and optimizing surveillance strategy of these malignancies in this specific subset of patients are an important facet of clinical care. For instance, primary sclerosing cholangitis is associated with an increased risk for cholangiocarcinoma (which is very challenging to diagnose) and when IBD is present for colorectal cancer. On the other hand, primary biliary cirrhosis patients with cirrhosis or not responding to 12 months of ursodeoxycholic acid therapy are at increased risk of hepatocellular carcinoma. In this review we will discuss in detail the risks and optimal surveillance strategies for patients with primary biliary diseases.

## 1. Introduction

Primary biliary diseases encompass several entities including primary biliary cholangitis (PBC), primary sclerosing cholangitis (PSC), autoimmune hepatitis (AIH), and overlap syndrome (classified as having PBC, PSC, or AIH). Patients affected with these diseases often present with a cholestatic biochemical profile and often are asymptomatic. Distinction between these entities is necessary due to variance of associated complications and recommended management. Complications of biliary diseases include an increased risk for development of malignancy especially in certain subsets of patients. In this review we intend to shed a light on advances in diagnosis and management of malignancy in patients with primary biliary diseases.

## 2. Overlap Syndromes

The serologic and clinical characteristics of AIH may overlap with other forms of chronic immune-mediated liver disorders such as PBC and PSC. Although the prevalence of overlap syndromes is small, it may affect the management and prognosis of patient's illness. The International AIH Group (IAIHG) recommends that patients with suspected overlap syndrome be classified on the basis of their primary disease as AIH, PBC, or PSC and therapy of primary disease should determine therapy [1]. A synopsis of the PBC-AIH and PSC-AIH overlap syndromes is as follows.

### 2.1. PSC-AIH Overlap

On the basis of criteria deemed arbitrary by the IAIHG, the frequency of PSC-AIH overlap ranges from 6% to 11% [2]. The hallmark of this overlap syndrome is the serologic finding reflective of AIH (presence of ANA and/or ASMA) and radiographic finding reflective of PSC. One study reported that PSC-AIH overlap patients had higher serum globulins (*P* = 0.01), IgG levels (*P* = 0.001), autoantibody titers (*P* < 0.001), and histologic scores (*P* < 0.001) than patients with PSC alone [3]. If a patient with known inflammatory bowel disease (particularly, ulcerative colitis) presents with pruritus, has an elevated alkaline phosphatase, and demonstrates radiographic or histologic findings of PSC, a diagnosis of PSC-AIH overlap can be considered. This overlap syndrome is difficult to treat. Although the safety and efficacy of immunosuppressive treatment are established in AIH, no effective therapy exists for PSC. A combination of immunosuppression (such as azathioprine) and varying doses of ursodiol may be tried; one study reported the efficacy of this regimen to be 20%–100% with inverse relationship to the severity of cholestasis. In selected patients, empiric cyclosporin, mycophenolate mofetil, and budesonide were also found to be beneficial [2].

### 2.2. PBC-AIH Overlap

One study reported the frequency of this overlap syndrome to be 7%–13% and reported the patients to be highly susceptible to a variety of autoimmune and immunologic diseases [2]. Two varieties of PBC-AIH overlap exist, based on presence or absence of antimitochondrial antibodies (AMAs). The AMA-positive variety of PBC-AIH overlap demonstrates histologic characteristics of AIH and responds well to steroid therapy. On the other hand, the AMA-negative variety of PBC-AIH overlap is usually positive for antinuclear antibodies (ANA) and/or antismooth muscle antibodies (ASMA) and has histologic findings and therapeutic management consistent with PBC. The AMA-negative variety of PBC-AIH overlap has also been termed “autoimmune cholangitis” or “immune cholangiopathy” and could also be considered as an AMA-negative form of PBC. The diagnostic criteria of the PBC-AIH overlap have garnered significant interest among gastroenterology circles. One of such diagnostic criteria, termed Paris criteria, requires the presence of two out of three diagnostic criteria for each of PBC and AIH to denote a case as PBC-AIH overlap. Accepted diagnostic criteria of PBC and AIH under Paris criteria are as follows:

Accepted criteria for diagnosis of PBC:

1. Twofold elevation in the alkaline phosphatase or fivefold elevation in the *γ*-glutamyl transferase.
2. Positive AMA.
3. Liver biopsy with bile duct lesions as seen in PBC.

Accepted criteria for diagnosis of AIH:

1. Elevated ALT of at least fivefold the upper limit of normal.
2. At least a twofold increase in IgG level or a positive ASMA.
3. A liver biopsy with classic findings suggestive of AIH.

Paris criteria yield high diagnostic accuracy with sensitivity and specificity of 92% and 97%, respectively [4].

PBC-AIH overlap syndrome responds well to a combination of immunosuppression (such as azathioprine) and ursodiol [1, 2, 4]. We learn the following lessons:

- Overlap syndrome could be classified as encompassing AIH and either PBC or PSC.
- AMA-positive AIH-PBC overlap demonstrates histologic characteristics of AIH and responds well to steroid therapy.
- AMA-negative variety of AIH-PBC overlap is positive for ANA and/or ASMA and should be managed as PBC.
- AIH-PSC overlap is difficult to treat.

## 3. Primary Sclerosing Cholangitis

Primary sclerosing cholangitis (PSC) is a cholestatic liver disease often affecting men (male : female, 2 : 1) in their fifties and is commonly associated with inflammatory bowel disease (IBD) [5, 6].

The pathogenesis of PSC remains obscure and is thought to involve (similar to PBC) several pathways that include autoimmune, genetic, and infectious processes. Evidence for an autoimmune component is underlined by its association with many extrahepatic autoimmune diseases. On the other hand, GWAS studies have revealed several genetic loci associated with an increased risk for development of PSC.

### 3.1. Risk of Malignancy

PSC is considered a premalignant condition. Risk for cholangiocarcinoma (CCA) and colon cancer and in cirrhotic patients risk for hepatocellular carcinoma are increased. CCA accounts for about 15% of primary liver and intrahepatic bile duct cancers annually in the United States with an incidence of 1-2 per 100,000 persons [7–9].

In a Swedish study by Bergquist et al., 44% of deaths in their cohort of patients were related to malignancies. It was determined that in their cohort of 604 PSC followed for 5.7 years the incidence of hepatobiliary carcinoma was 1.5% per year and cholangiocarcinoma was 13%. Most importantly, pancreatic carcinoma risk was found to be 14 times higher compared with the general population and malignancy can affect up to 25% of the patients with PSC [10].

The presence of dominant stenosis/strictures (defined as a stricture less than 1.5 mm diameter in the common bile duct or less than 1 mm in the left or right main hepatic ducts) when accompanied with IBD seems to be associated with an increased risk of cancers including biliary, gallbladder, and colorectal malignancies as compared to those without preexistent IBD. This may suggest that patients with dominant stenosis may represent a sicker group of people with worsened outcomes [11].

There is a widely accepted sequence in development of CCA in PSC inflammation-dysplasia-carcinoma. Neither of other suggested predictors as elevated Bilirubin, variceal bleeding, older age, and duration of IBD was found to be clinically useful. CCA develops independently of cirrhosis. A study performed at the Mayo Clinic demonstrated that inducible nitric oxide (iNOS) is expressed in PSC and CCA but not in normal biliary epithelium and increases in 8-oxodeoxyguanosine. These findings with generation of oxysterols in bile support inflammatory theory of pathogenesis [12]. Signaling has been implicated in biliary cancers such as CCA in patients with PSC [13–17]. Genetic polymorphism of natural killer cell receptor G2D (NKG2D) is seen to be a risk factor for these patients [18], as well as loss of CDKN2A/p16 gene at the chromosome 9p21 locus which is a marker for dysplasia [19].

PSC is the most common risk factor for developing CCA in the Western countries [20]. CCA can be found in 5–15% of patients with PSC [21, 22] with the annual incidence rate of 0.6–1.5% and lifetime risk of 7 to 20% [5, 23]. The prognosis for CCA is very poor with median survival time of 5 months after diagnosis [24].

A study from Finland utilizing 20 years of follow-up reports almost 1000-fold risk of CCA in concomitant IBD and PSC compared to the general population [25]. More than a third of the patients with PSC are expected to be diagnosed with CCA within the first year of having PSC, with most CCAs being found in the first 2.5 years after the conclusion of PSC [23]. Furthermore, a study evaluating autopsy findings estimated the occurrence of CCA in patients with PSC at 40% [26, 27].

Due to lack of sensitive diagnostic testing, distinction between benign bile duct strictures and CCA remains challenging. Serological testing is of importance in making that distinction. Tumor marker CA 19-9 with a serum level higher than 100 IU/mL has 75% sensitivity and 80% specificity in identifying CCA in patients with PSC. Accuracy can be improved to 86% by addition of CEA [28]. Increasing the CA 19-9 cut-off level to >129 IU/mL in one study has improved specificity to 99% but only in the absence of bacterial infection [29].

Combining serological and imaging techniques may yield increased sensitivity and specificity for diagnosis of CCA in PSC. In an observational study aiming at evaluating the role of combinations of serological testing and imaging in detecting CCA in patients with PSC it was determined that CA 19-9 combined with one of ultrasonography, computed tomography, or magnetic resonance imaging provided a sensitivity of 91%, 100%, and 96%, specificity of 62%, 38%, and 37%, PPV of 23%, 22%, and 24%, and NPV of 98%, 100%, and 98%, respectively [30].

It is worth noting that elevated serum biomarker may carry a prognostic utility following tumor resection. Such utility has been demonstrated in patients with pancreatic adenocarcinoma where CA 19-9 was predictive of postresection survival [31].

Nonetheless, bile duct brushings during ERCP remain first-line investigative procedure for biliary strictures, which also helps palliate dominant strictures. However, sensitivity yield remains low at around 40–50%. American Society of Gastrointestinal Endoscopy recommends > 5 passes across the stricture, removal of brush and catheter together, and inclusion of washing into the specimen to increase diagnostic yield of the test [32]. Cleveland Clinic uses two sets of brushings: one for cytology and one for FISH [33]. One small sized Japanese study reports achieving 100% specificity with forceps biopsy [34].

Fluorescence in situ hybridization (FISH) is used to reveal chromosomal abnormality by fluorescence-labeled probes. There are four commercially available FISH probes which bind to chromosomes 3, 7, 17, and 9p21 locus, responsible for p16 tumor suppressor gene. Obvious advantages of FISH as compared to endoscopic testing are the lack of interobserver variability and simplicity. One of the main concerns regarding the application of FISH in early detection of CCA in the patients with PSC is the modest sensitivity and can be considered exclusively in patients with a high pretest probability [35].

A meta-analysis conducted by Navaneethan et al. found that pooled sensitivity and specificity for FISH test using polysomy alone as a positive result for the diagnosis of CCA in PSC patients were 51% (95% CI: 43%–59%) and 93% (95% CI: 91–95%), but low likelihood ratios (positive at 6.51 and negative at 0.56) do not allow using FISH as a single test in diagnosing CCA with PSC patients [36]. Patients with CCA associated with PSC have higher (80%) prevalence of DNA aneuploidy than those with PSC and without CCA [37].

Eaton et al. further evaluated the opportunities provided by fluorescence in situ hybridization [38]. They concluded that multifocal polysomy detected by the FISH in multiple areas of biliary tree is the strongest predictor of CCA diagnosis among PSC patients suspected of having biliary cancer.

A recent analysis of biliary brush samples for DNA methylation of certain genes identified four genes: CDO1, CNRIP1, SEPT9, and VIM. Use of these genes as a panel displayed 85% sensitivity and 98% specificity in early detection of CCA in PSC patients [39].

One of the last trends in noninvasive diagnosis of malignant biliary strictures is measurement of “Volatile Organic Compounds” in bile or urine. A study from Cleveland Clinic reported the utility of ethane, 2-propanol, trimethylamine, carbon disulfide, and 1-octene levels as predictors of biliary malignancy in PSC patients [40].

Another interesting approach in diagnosing CCA in patients with PSC is the analysis of miRNA patterns in serum and bile. Several miRNAs occur at lower concentrations in CCA compared to PSC patient without CCA. The most promising miRNA in the serum in this regard was miR-126 with specificity of 93% [41].

Another technique is analysis of bile and serum peptides with capillary electrophoresis coupled with mass spectrometry. This allows for discrimination between PSC with CCA and absence of CCA with 84% sensitivity [42]. Lankisch et al. propose using urine to obtain bile instead of utilizing invasive and time-consuming endoscopic procedures.

Novel diagnostic modalities have been introduced recently; these, however, carry a disadvantage of the results being operator-dependent. One such modality is intraductal ultrasonography (IDUS) which can be performed as a part of routine ERCP without the need of sphincterotomy. This technique, however, does not provide histopathology limiting its use to an adjunct diagnostic tool despite its reasonably good sensitivity and specificity, especially in the proximal biliary strictures [43].

On the other hand, cholangioscopy has a specificity of 82–90% which is even higher with visual targeted biopsy [33, 44, 45]. However, the value of the visualization without clinical correlation limits the utility of this method for surveillance of dominant stenosis [43, 46]. Another limiting factor is the associated complications related to the need of performing a sphincterotomy to conduct the test. Serious procedure-related adverse events of cholangioscopy have been reported at around 7.5% [44]. Other complications may occur with tight distal strictures where the rate of postprocedural cholangitis is reported at 11% [47].

Narrow Band Imaging is another modality which provides improved visualization. One study reported increased detection of suspicious lesions which was not confirmed by dysplasia detection [48]. Probe based confocal laser endomicroscopy (pCLE) detects neovascularization and abnormal vessels in biliary strictures. This technique has been proposed as a method with high technical success in patients with PSC for exclusion of CCA with high sensitivity and negative predictive value [49]. This method, however, has a reported specificity of only 61%. However, introduction of the Miami classification and Paris inflammatory criteria may help improve specificity [50]. Meining et al. described an increase in accuracy by addition of pCLE to ERCP when compared to ERCP and tissue sampling alone [51]. This may also carry the advantage of reducing the frequency of tissue samplings in patients with PSC and dominant strictures during evaluation for CCA. Widespread use of this technology is limited by the need for specialized operator training and low specificity [46].

### 3.2. Surveillance

Diagnosis of CCA in PSC may be very challenging and may sometimes require rather invasive techniques as discussed above. Hence, no accepted surveillance strategy for CCA in PSC is currently present.

Because sensitivity and accuracy of the biliary brushings with FISH are still debatable and these tests may carry certain risks for complications from ERCP (such as pancreatitis and cholangitis) this method is not recommended for surveillance at this point but is very useful for diagnosis.

Recommendations for offering testing of liver enzymes every 6 months with an annual check of CA 19-9 plus any of the imaging studies available (MRCP, US, and CT cholangiography) have been previously suggested [20]. In the cases of dominant stenosis or any suspicion indicating the need to proceed with ERCP, bile brushings and FISH are anticipated.

### 3.3. Colorectal Cancer

Colorectal cancer (CRC) is annually diagnosed in 134,490 men and women in the United States [52]. Some early studies suggested that the prevalence of CRC in the setting of concomitant PSC and ulcerative colitis (UC) is around 9–14% in the first 10 years of establishing a combined diagnosis, 31% at 20 years, and up to 50% at 25 years [53].

In a study looking at risk of cancers in patients with PSC and inflammatory bowel disease, the authors reported an increased risk for CRC with odds ratio of 5 (95% CI: 2.80–8.95) [54]. The finding of increased risk for CRC in PSC-UC patients was also reported in a meta-analysis of eleven studies [55].

Hence, current guideline recommendations [5] are to perform screening colonoscopy with biopsy at diagnosis of PSC and then every 5 years if no IBD is present and yearly if IBD is present [53, 56–60].

### 3.4. Gallbladder Carcinoma

Gallbladder cancer has an incidence of 1 to 2 cases per 100,000 persons in the US [61]. Patients with PSC are at increased risk of gallbladder carcinoma. A study of 102 PSC patients undergoing cholecystectomy revealed a 13.7% occurrence of gallbladder lesions with greater than 50% of these lesions being malignant [62]. Hence, current guidelines recommend annual surveillance for gallbladder lesions with U/S [5].

### 3.5. Hepatocellular Carcinoma

A German study aimed at evaluating the risk of HCC in more than 500 PSC patients did not show increased risk of HCC in this subset of patients [63]. We learn the following lessons:

- PSC carries an increased risk for cholangiocarcinoma (CCA) and colon cancer and in cirrhotic patients for hepatocellular carcinoma.
- The annual incidence rate of CCA is reported to be 0.6–1.5% with a lifetime risk of 7 to 20%.
- Diagnosis of CCA in PSC may is very challenging.
- PSC-UC involves a dramatic increased risk of CRC.
- Perform screening colonoscopy with biopsy at diagnosis of PSC and then every 5 years if no IBD is present and yearly if IBD is present.

## 4. PBC

PBC is a rare chronic cholestatic liver disease that, if left untreated, eventually culminates in cirrhosis and liver failure. The exact pathogenesis of PBC remains under investigation. One of the proposed mechanisms includes the obliteration of small intralobular bile ducts through T-lymphocyte-mediated activity. Other proposed mechanisms involve environmental, geographic, and genetic factors.

Evidence for underlying autoimmune disease is supported by the presence of circulating antibodies and elevated immunoglobulins on serology, association with other autoimmune conditions, and development of granulomas in patients with PBC.

Environmental factors have been implicated by studies such as the one published by Prince et al. and McNally et al. that sought to describe the temporal and spatial distribution of PBC within defined geographical areas. PBC was found to be significantly more prevalent in urban areas when compared to rural locales [64]. Moreover, areas with higher levels of socioeconomic deprivation had an increased risk of PBC (*P* = 0.035) in McNally's study. Spatial clustering of PBC cases was also confirmed in this study [65].

Additionally, Muirhead et al. discovered that PBC demonstrated a temporal pattern suggesting a possible role of seasonal factors affecting the disease [66].

Genome Wide Association Studies (GWAS) in PBC have further solidified a role of a genetic component and have been instrumental in advancement of our knowledge in the pathogenesis of PBC. According to a review by Gulamhusein et al., there have been six large scale studies which have identified 27 risk loci in addition to HLA associated with PBC [67]. Two such studies published by Underhill et al. revealed an association between PBC and human major histocompatibility complex (HLA) DR8 and DPB subgroup [68, 69]. Another study published by Wang et al. described increased frequency of circulating T follicular helper (Tfh) cells in PBC patients. Additionally, this study described a decrease in Tfh cells in patients using ursodeoxycholic acid (UDCA) [70]. This study was reinforced by Limongi's findings which demonstrated a significant reduction of T-helper 1 cytokines after treatment with UDCA [71].

Some studies also indicate that infectious processes may play a role in the progression of PBC. Some of the implicated infections include* Chlamydia pneumoniae*,* E. coli* (particularly UTI caused by* E. coli*), and* Lactobacillus*. To further establish an infectious role to the progression of PBC, Thomas et al. demonstrated that zidovudine was associated with a significant reduction in alkaline phosphatase as well as cholangitis and ductopenias at 12 months. Other studies detailed in that same paper described promising trials using the combination of tenofovir and emtricitabine and lopinavir [72].

### 4.1. Risk of Malignancy

Several studies have indicated that patients with PBC are at increased risk for specific malignancies such as hepatocellular carcinoma.

Cirrhotic PBC patients are at increased risk of hepatocellular carcinoma (HCC). A study in the Greek population of PBC patients revealed a 10-year risk of 4% for developing HCC (15% in cirrhotic patients) and of 13% for developing extrahepatic malignancies [73]. A similar study in Spanish and Italian populations revealed that the prevalence and incidence (0.35 and 0.37 per 100 patient-years in Barcelona and Padova accordingly) of HCC were similar. Only advanced histological stage was associated with around a sixfold risk of development of HCC (odds ratio [OR]: 5.80, 95% confidence interval [CI]: 2.34–14.38, *P* < 0.001). On the other hand, male gender, age >52 years, smoking, alcohol >40 g/day, presence of HBsAg, and anti-HCV were not associated with HCC [74]. Unlike cryptogenic and alcoholic cirrhosis, obesity does not appear to be an independent risk factor for development of HCC in PBC patients [75].

A recent study by Boonstra et al. involving 992 PBC patients followed for a median of 73 months (range: 0–434) concluded that there was a ninefold increased risk of developing hepatobiliary malignancies (incidence ratio: 9.4; 95% CI: 3.04–21.8) [76]. The risk for developing HCC was also confirmed in a 2012 meta-analysis by Liang et al. which revealed that patients with PBC have a relative risk of 19 (95% CI: 11–27) as compared to the general population for developing HCC [77].

A recent international multicenter study revealed that 12-month biochemical nonresponse in patients with PBC on ursodeoxycholic acid was associated with increased risk of developing HCC [78].

Conflicting evidence regarding increased risk of breast cancer or lack thereof has been published [79–83]. On the other hand, Boonstra et al. revealed that patients with PBC have a fivefold increase in risk for developing urinary bladder cancer (SIR 5.0; 95% CI: 1.6–11.6) and 1.8-fold increase in risk for developing breast cancer (SIR 1.8; 95% CI: 1.0–2.81) [76]. We learn the following lessons:

- Cirrhotic PBC patients are at increased risk of hepatocellular carcinoma (HCC).
- 12-month biochemical nonresponse in patients with PBC on ursodeoxycholic acid was associated with increased risk of developing HCC.
- There is conflicting evidence regarding increased risk of breast cancer in PBC patients.

### 4.2. AIH

AIH is characterized by high globulin levels, autoimmune features, and circulating antibodies directed against self; AIH is a chronic inflammation of the liver that can progress to cirrhosis. Due to the variety of ways AIH can manifest, various immunogenic phenotypes, circulating autoantibodies, and clinical features have been used to characterize the disease process. Among the modalities used to describe this disease process, classification using circulating autoantibodies has been suggested but has not been that effective given the lack of evidence that ties these antibodies to the pathogenesis of AIH.

One of the major theories for the pathogenesis of AIH proposes a combination of environmental triggers (which includes viruses, herbal supplementations, medications, and immunizations) in a patient who is genetically predisposed. Despite lack of evidence regarding detailed associations between antigens, genetic predisposition, and the autoimmune process, the biomolecular level is thought to involve interaction between antigen, MHC, and T-cell receptors forming a complex that serves as a contact point to induce autoimmunity. However, the exact inducers of autoimmunity cannot be specified. It is also reported that change in T-cell function plays a central role in the pathogenesis of AIH with loss of tolerance via absence of normal suppression of self-reactive T-cells, with B cell abnormalities playing a lesser role. This mechanism of loss of tolerance contributes to repetitive inflammation and necrosis of liver in AIH. The immunoglobulin superfamily which also include HLA class within the MHC, immunoglobulins, and T-cell receptor molecules have been the targets with ongoing research to identify genetic predisposing factors. The HLA-DR3 serotype has a strong presence in Caucasians with type I AIH with early onset and severe disease, while the HLA-DR4 serotype is more prevalent in Caucasians with late-onset disease. HLA-DR4 is also associated with higher rates of extrahepatic manifestations and improved steroid responsiveness.

The hallmark features of AIH are represented by its immunologic and autoimmunologic features in the setting of circulating autoantibodies and hyperglobulinemia. Despite the lack of convincing evidence establishing the role of these antibodies in the pathogenesis of AIH, 2 major forms of AIH have been proposed based on these immunologic features: type I AIH and type II AIH.

Type I AIH, also known as classic AIH, is characterized by antinuclear antibodies (ANA), antismooth muscle antibodies (ASMA), and IgG Actin (AAA). According to Frenzel et al., F-actin ELISA had superior sensitivity (100%) and similar specificity (98%) for diagnosis of AIH compared with the standard antismooth muscle antibody immunofluorescence testing [84]. Atypical p-ANCA, anti-SLA/LP (soluble liver antigen/liver pancreas antigens), and double-stranded DNA are some autoantibodies known to occur in type I AIH. Type II AIH is characterized by antibodies to liver/kidney microsomes (ALKM-1) and to liver cytosol antigen (ALC-1).

The mainstay of treatment for AIH is prednisone with or without azathioprine [85]. Various factors play a role in the relapse of AIH including lack of response to medication, intolerance to medication due to side effects, or disease recurrence after completion of course of treatment. A case report described two patients with AIH refractory to standard treatment with the first patient requiring tacrolimus, mycophenolate mofetil, and budesonide to achieve remission. The second patient required rituximab as a replacement for sirolimus with an addition of mycophenolate mofetil and prednisone to achieve remission [86]. In a single-center study examining six patients with biopsy proven AIH refractory to prednisone and azathioprine, two infusions of rituximab 1000 mg two week apart led to biochemical improvement without serious side effects [87].

### 4.3. Risk of Malignancy

Among the various manifestations of AIH, hepatic and extrahepatic malignancies are present throughout the course of the disease in patients with AIH. Malignancies can arise secondary to the underlying disease process, appear independent of AIH, predispose to AIH, and occur due to prolonged immunomodulation therapy for AIH [88–94].

Hepatocellular carcinoma (HCC) is a known outcome in patients with AIH and cirrhosis with cirrhosis being a requirement for developing HCC [95–99]. Patients with AIH with the highest risk for HCC have certain defining features including cirrhosis for more than 10 years, portal hypertension and its sequelae, repetitive liver inflammation, and immunosuppressive therapy for more than 3 years [95, 96, 98–100]. In a study looking at the risk Yeoman et al. [97] established that HCC arises more frequently in AIH patients with cirrhosis at presentation (9.3% versus 3.4%, *P* = 0.048). Thus, cirrhosis in AIH appears to be a prerequisite for HCC development, which consequently arises at a rate of 1.1% per year and equally affects males and females. Another study reported an incidence rate of 0.3% cases per year of follow-up after the development of cirrhosis in AIH patients [101].

Hematogenous metastasis of hepatocellular carcinoma to the ascending colon in a patient with AIH has also been documented in a case report [102]. In another case report, gastric adenocarcinoma occurred after cadaveric liver transplantation in a patient with AIH; the exact role of the AIH in the development of the gastric cancer was not elucidated [103]. Just as AIH is suspected to contribute to the development of gastrointestinal cancers, it can also develop in the setting of a gastrointestinal malignancy. A case review asserted that AIH occurred de novo in 5 patients with hematologic malignancy and in 1 patient with colon cancer. The AIH occurred as an overlap with PSC and PBC in two of the cases. However, the review could not determine whether AIH developed due to the underlying disease itself or due to the cancer treatment [104]. We learn the following lessons:

- Immunogenic phenotypes, circulating autoantibodies, and clinical features have been used to characterize AIH due to its various manifestations.
- The mainstay of treatment for AIH is prednisone with or without azathioprine.
- HCC occurs more frequently in AIH patients with cirrhosis and may occur at a rate of 1.1% per year.
